# An ultrafast quantum thermometer from graphene quantum dots

**DOI:** 10.1039/c8na00361k

**Published:** 2019-03-06

**Authors:** Poonam Sehrawat, Abid, S. S. Islam

**Affiliations:** Centre for Nanoscience and Nanotechnology, Jamia Millia Islamia (A Central University) New Delhi 110025 India sislam@jmi.ac.in +91 11 26987153

## Abstract

We report an ultra-sensitive temperature sensor derived from graphene quantum dots (GQDs) embedded in a self-standing reduced graphene oxide (RGO) film. The GQDs are obtained as a natural derivative during synthesis of GO to RGO. A fundamental study on low temperature transport mechanisms reveals the applicability of temperature zone specific ‘variable range hopping (VRH)’ conduction models, *i.e.* Mott-VRH, Efros–Shklovskii-VRH and activation energy supported VRH. On the basis of transport behavior and confirmed by characterization analyses, the RGO film is modeled as GQD arrays where graphitic (sp^2^) domains behave as QDs and oxygenated (sp^3^) domains between interdots act as tunneling barriers. Temperature dependent resistance and current–voltage (*I*–*V*) characteristics indicate high sensitivity where sensor resistance changes by almost six orders of magnitude as the temperature is varied between 300 and 12 K. In convection mode, the developed temperature sensor shows a temperature coefficient of resistance (TCR) of ∼−1999% K^−1^ in the 300–77 K temperature range, which is much higher than the TCR values reported so far. Additionally, the sensor exhibits an extremely fast response (∼0.3 s) and recovery (0.8 s) time; and such high TCR leads to ultra high resolution of ∼ μK. The sensor shows excellent repeatability with negligible drift over several cycles. These studies are crucial for modern day thermal management and sensitive cryogenic applications.

## Abstract 2

We report an ultra-sensitive temperature sensor derived from graphene quantum dots (GQDs) embedded in a self-standing reduced graphene oxide (RGO) film.

## Graphical abstract

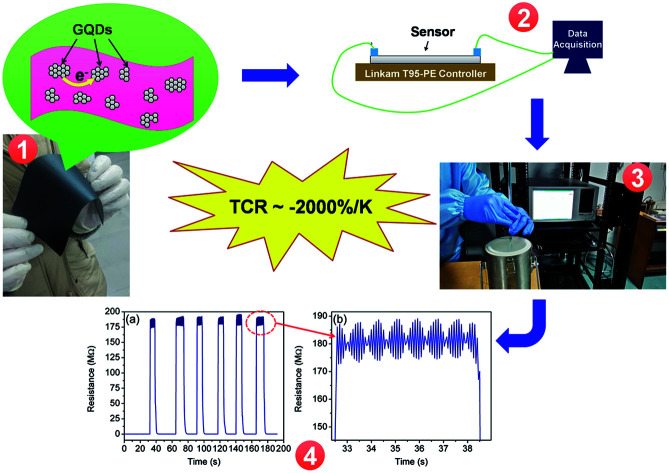

## 1. Introduction

Since its inception, carbon nanotechnology has opened new prospects for the synthesis and analysis of one- and two-dimensional materials wherein fundamental physics converges with device applications.^1–5^ One such material is graphene, whose electronic applications have been precluded by the zero bandgap at the charge neutrality point which thwarts its use in logic devices requiring frequent switching between ON/OFF states.^6–17^ This limitation can be overcome by modifying the band structure of graphene *via* lateral quantum confinement in quantum structures such as quantum dots (QDs).^18–21^ Since the initial studies on QD physics in the 1980s, QDs have continuously gained zealous efforts from the research community. Irrespective of the dot size, graphene quantum dots (GQDs) possess distinctive graphene structures inside the dots which endows them with some of the remarkable properties of graphene;^22–26^ rendering them ideal systems for observing a novel quantum phenomena and building blocks for futuristic electronic devices in sensor applications.^24,26,27^ Nonetheless, the handling and device fabrication pose challenges, thereby necessitating the search for alternative graphene like materials which are easy to fabricate and possess properties close to graphene. An enormous quality research study is underway to search for such materials with mass production and ease of device fabrication.

The most successful routes to synthesize such materials rely on using graphite as the precursor.^28–32^ One such route is a chemical synthesis technique where exfoliated graphene oxide (GO) is produced in the first step and then reduced to obtain reduced graphene oxide (rGO).^33–36^ This technique is most suitable for economic and large scale synthesis of rGO. Despite having lowered electrical and thermal properties in comparison to pristine graphene, rGO is interesting owing to its unique properties which are an amalgamation of properties similar as well as dissimilar to those of pristine graphene.^37,38^ Various functional groups, such as epoxy (–O–) and hydroxyl (–OH) covalently attach on the basal plane, whereas carboxylic (–COOH) and carbonyl (–C=O) groups primarily append at the sheet edges.^18^ Besides, sp^3^ → sp^2^ carbon transformation in rGO is not hundred percent complete and causes considerable defects or disorders which can trap charge carriers.^39^ Additionally, the conductivity of an rGO film can be controlled by varying the film thickness which in turn depends upon the synthesis parameters (*e.g.*, ultrasonication, reduction time, *etc.*).^3^ Thus, rGO has gained immense scientific interest due to – (a) large scale production; (b) modifiable properties which can be controlled by tuning the sp^2^ to sp^3^ carbon ratio; and (c) a possibility of further modifying the rGO structure (and hence its properties) *via* functionalization with different nanoparticles as well as organic molecules.^40^ This behavior is attributed to the presence of significant disorders on the rGO films. Thus, a comprehensive study needs to be undertaken to analyze the effect of synthesis conditions on all these parameters.

Interestingly, much of the initial work on graphene based devices, in general, has been on field effect transistors (FETs). Development of a smart temperature sensor from graphene is a relatively new area of research.^41–43^ Although a substantial amount of work has been done on the carrier transport mechanism in graphene,^17,38,44–48^ hardly any studies are available on the development of temperature sensors either from graphene or GQD arrays, exploiting their carrier transport properties. In the case of carrier transport in graphene or 2D materials, the current flow is controlled by scattering events;^49–52^ whereas in GQDs, the current will exist only if carriers overcome the Coulomb blockade potential.^53,54^ The temperature dependent differential change in resistance/current in the circuit is the key factor for the development of a highly sensitive temperature sensor. Two major sensing parameters, *i.e.* temperature coefficient of resistance (TCR) and thermal hysteresis (*H*_Th_) are very crucial since both of them control the quantification of heat sensing characteristic parameters including sensitivity, response- and recovery-time, resolution and repeatability. Interestingly, not many reports are present on GQD based temperature sensors and no concrete report is presented so far.

In this article, we present the rich transport phenomena observed in the rGO/alumina self-standing film where the resistance can be tuned over more than six orders of magnitude *vis-à-vis* its room temperature value. The rGO film has been converted into nanometre sized graphitic zones (termed as graphene quantum dots or GQDs) caged within insulating zones (barrier). The GQDs, distributed in disordered arrays, demonstrate elevated temperature (*T* ∼ 300 K) activated hopping, and direct quantum tunneling depending on the spatial interdot separation and operating temperature. The temperature sensor fabricated from this film shows TCR values of −7946.5% K^−1^ and −1999.8% K^−1^ in the temperature ranges of 300–12 K and 300–77 K, respectively. Such a high TCR leads to the achievement of ultra-resolution of ∼ μK, and a response-and recovery-time of 0.3 s and 0.8 s respectively in convection mode. Cycling tests conducted at 77 K show negligible drift over several cycles. Additionally, this work presents an example of reversible metal–insulator transition in a solid-state system implying the prospects of producing novel solid-state materials, based on GQDs, wherein the electronic band structure of the material can be ‘tailored’ by adjusting the electronic wave function overlap between neighboring dots.

## 2. Materials and methods

### 2.1. Materials synthesis

Transformation of graphite to graphene oxide (GO) *via* a chemical conversion technique has surfaced as a convenient method to produce graphene like monolayers with considerable yield.^51,52,55^ Modified Hummers’ method based oxidative treatment of natural graphite flakes was performed to prepare graphite oxide.^56^ Unlike pristine graphite, GO is highly oxidized having oxygenated epoxide bonds on the basal plane, whereas carbonyl and carboxyl groups are present at the sheet edges. Single- to few-layered GO sheets were obtained by exfoliation of graphite oxide in DI using a bath sonicator (90 minutes). The presence of oxygen moieties makes GO sheets readily soluble in DI, resulting in stable aqueous suspensions of nearly 1 nm thick sheets under mild sonication conditions.^57^ GO was then washed and dried under ambient conditions. Chemical reduction of the GO powder was carried out by dissolving 500 mg GO in 500 mL DI followed by 1 h of sonication. To this mixture, 50 μL hydrazine hydrate was mixed and the solution was refluxed at a constant temperature (98 °C) for varying time periods (1, 2, 3, and 5 h). At this point, the color changed from yellowish brown to black, indicating the conversion of GO to rGO. The RGO powder was obtained by suction filtering and washing the suspension with DI followed by subsequent drying at 60 °C in an inert (N_2_) ambient atmosphere for 10 h.^51,52^ The reduction process removes epoxy groups from the basal plane resulting in regeneration of C–C bonds.^55^

To understand the mechanism of the above reactions, we can consider a graphene lattice,^58^ where three out of four valence electrons from each C atom are covalently bound to three neighboring C atoms. These bound electrons are called σ-electrons and the corresponding bonds are termed as σ-bonds or C–C bonds. The fourth electron from each C atom (termed as π-electron) is free to move around the graphene layer, and thus acts as a charge carrier. In the hexagonal lattice of graphene, these π-electrons occupy six individual corners of the first Brillouin zone (also called the Dirac points).^58^ During oxidation, each oxygen (O) atom uses two π-electrons from two C-atoms and meets its octet requirements, thereby forming epoxy bonds. However, neither σ-electrons nor σ-bonds are altered to fulfill octet requirements because the bond energy of C–O (358 kJ mol^−1^) is much lower *vis-à-vis* C–C (473 kJ mol^−1^) in graphene.^58–60^ Four types of oxygenated functional groups exist in GO, these are^61^

(a) Epoxide (–O–): attached to the basal plane.

(b) Hydroxyl (–OH): along the basal plane.

(c) Carbonyl (–C=O): distributed along the edges.

(d) Carboxyl (–COOH): attached to the edges.

Out of these, epoxide and hydroxyl groups dominate the total oxygen functionalities glued to GO sheets.^38^ During reduction by a chemical route or thermal treatment, the C–O bonds break, thereby again freeing the π-electrons. In this way, graphene oxide recovers its pristine state by clearing the oxygen debris created during the oxidation process. Further, there was no apparent involvement of either σ-electrons or σ-bonds (or C–C bonds) unless aggressive oxidation/reduction is pursued. Thus, the graphene lattice is usually intact and no considerable point defects are introduced. Additionally, carbonyl and carboxyl groups cannot be removed without destroying the graphene basal plane.^62^

GO to rGO transformation leads to the creation of graphene quantum dots (GQDs) within the rGO maze and their sizes vary from a few nm to 100 nm depending on the degree of conversion from sp^3^ to sp^2^ bonding. The graphene region (sp^2^ bonding) shows semi-metallic behavior whereas the remaining sp^3^ region is insulating. Physical properties of rGO are considerably affected by the sp^2^/sp^3^ hybridized carbon ratio, and the presence of different functional moieties. Therefore, the electrical conduction process through the rGO sheet becomes complex and it shows the Coulomb blockade phenomena for charge transport, specifically at low temperatures.^23,55,63,64^ Growth of QDs in the rGO film have been studied by several groups,^23,63–66^ where the degree of reduction during the synthesis process and atomic scale features are studied and characterized with various microscopic and spectroscopic techniques.

### 2.2. Materials characterization

#### 2.2.1. XPS analysis

**Fig. 1:**
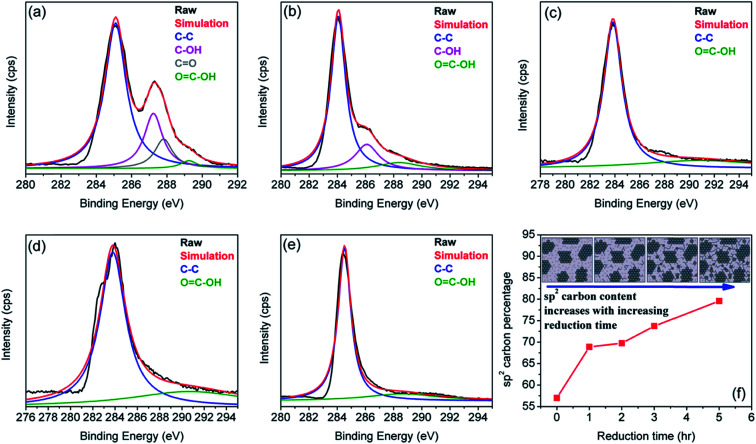
XPS spectra of the rGO sample for (a) 0 h (GO), (b) 1 h, (c) 2 h, (d) 3 h, and (e) 5 h of reduction treatment. (f) Shows the variation of sp^2^ carbon as a function of reduction time. The inset to (f) is a structural model of GO at various steps of the reduction process. Here, dark areas indicate sp^2^ carbon domains, and light grey areas are sp^3^ carbons consisting of oxygen groups (depicted as small dots). Reproduced with permission from ref. 18.

The changes in carbon and oxygen groups were analyzed by studying the gradual removal of oxygen containing functional groups by employing X-ray photoelectron spectroscopy (XPS). Since sp^3^ defects alter the intrinsic π state of sp^2^ sites,^35,55,62,67,68^ residual sp^2^ carbon fraction becomes a vital clue for rGO sheets and is often described as the reduction efficiency. Fig. 1(a–e) show deconvoluted C1's peaks in the XPS spectra of GO and rGO sheets at each reduction step. From Fig. 1(a), four individual peaks indicating different bonds of carbon atoms are readily perceived as: (1) the non-oxygenated C–C bond (284.5 eV ± 0.4 eV, blue curve); (2) the C–OH bond (286 eV ± 1 eV, magenta curve); (3) the C=O bond (287.3 eV, grey curve); and (4) the C(O)O or O=C–OH bond (289.2 eV ± 0.4 eV, green curve). Fig. 1(b–e) show the C1's spectra of rGO, where gradual attenuation and disappearance of some oxygenated groups are apparent. The C–C peak represents the sp^2^ carbon fraction, while the oxygenated functional moieties present at the basal plane of the sheets and sheet edges signify sp^3^-hybridized carbon fraction.^38,67,69,70^ The sp^2^ carbon fraction is obtained from the ratio of the integrated peak areas of the C–C peak to the total area under the C1's spectrum:^40^
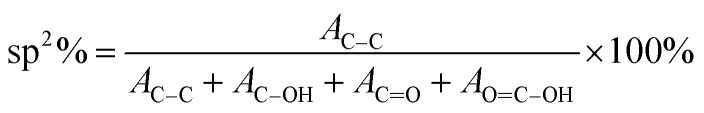


The results in Fig. 1(f) indicate that sp^2^ carbon fraction (or reduction efficiency) of the rGO sheet increases from ∼57% for GO (0 h) to ∼80% for rGO treated for 5 h.

#### 2.2.2. HRTEM studies

**Fig. 2:**
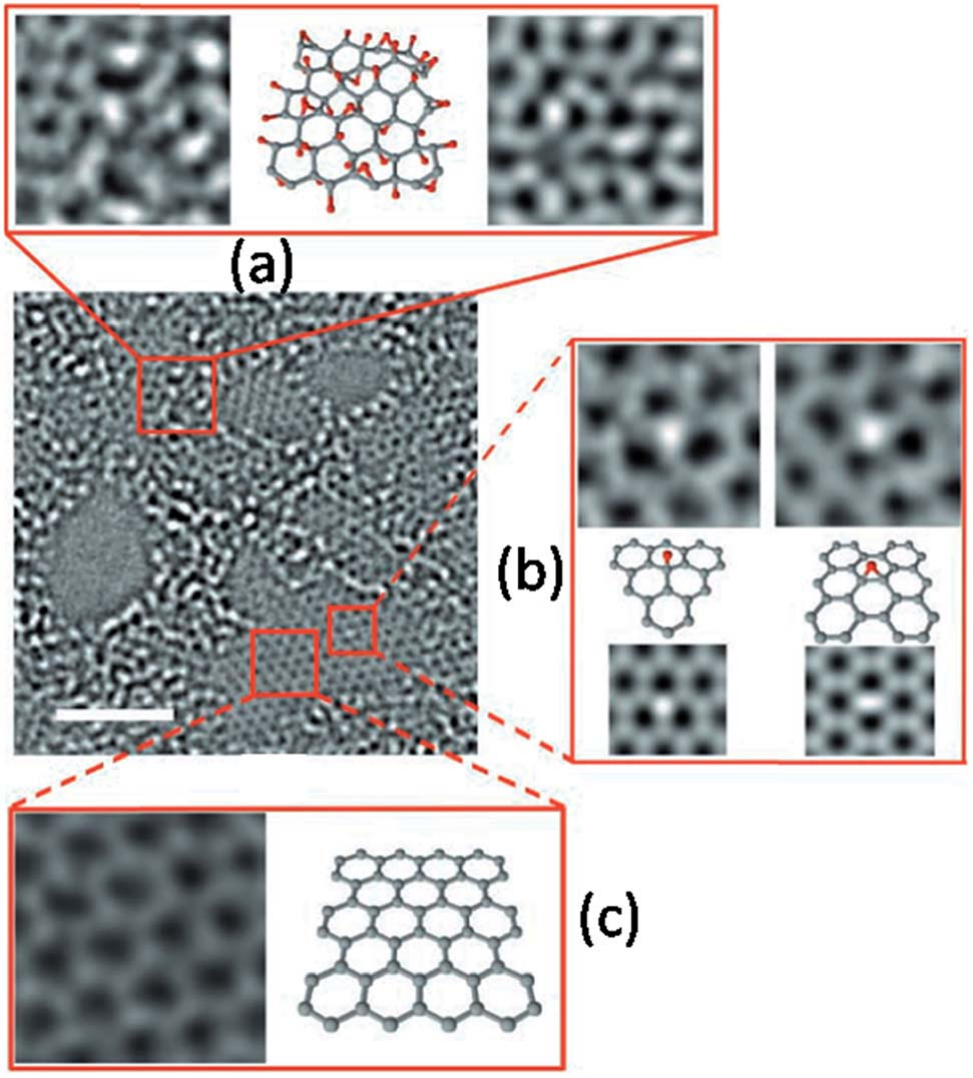
Aberration-corrected TEM images of a monolayer GO on a 2 nm scale. (a) Leftmost image shows the 1 nm^2^ enlarged oxidized region in GO, the middle image is its suggested atomic structure where grey dots represent carbon atoms and red dots are oxygen atoms, and the rightmost image is the average of a simulated TEM micrograph of the suggested structure and a simulated TEM image of a second structure with changed positions of oxygen containing groups. (b) Enlarged image of the white spot in the graphitic region. This spot moved along the graphitic region, but stayed stationary for three frames (6 s) at a hydroxyl position (left side of expansion (b)) and for seven frames (14 s) at a (1, 2) epoxy position (right side of expansion (b)). The ball-and-stick figures below the microscopy images show the proposed atomic structures for these functional groups. The simulated TEM image for the proposed structure is in agreement with the observed TEM structure. Enlarged image in (c) displays a 1 nm^2^ graphitic section from the exit plane wave reconstruction of a focal series of GO and the atomic structure of this region. Reproduced with permission from ref. 72.

As indicated in Fig. 1(f), the reduction process results in restoration of sp^2^ C–C bonds. Nevertheless, a continuous sp^2^ phase is not observed, rather these sp^2^ zones remain spatially separated by ‘islands’ of sp^3^ clusters. Thus, the reduction of GO creates numerous sp^2^ clusters of small size.^71^ This has been observed by Erickson *et al.* in ref. 72, and their observations are included in Fig. 2, where the graphitic sp^2^ zones appear to be surrounded by disordered sp^3^ domains. The size of these sp^2^ zones is found to vary from 2 to 6 nm.

#### 2.2.3. Raman spectroscopy

**Fig. 3:**
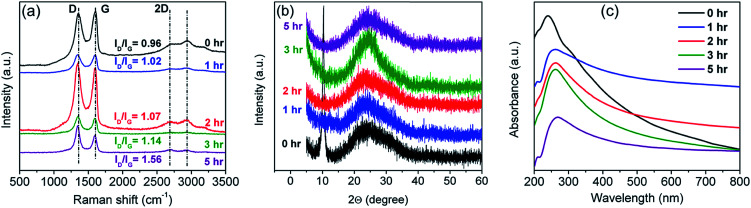
(a) Raman spectra, (b) XRD patterns, and (c) UV-Vis spectra of the prepared rGO sample as a function of reduction time.

The most remarkable peaks in Raman spectra of graphene and alternative graphitic materials comprise D, G, and 2D peaks at ∼1350 cm^−1^, 1580 cm^−1^, and ∼2700 cm^−1^ respectively.^38,73^ The D-band arises from disorders in atomic arrangement, edge effects, ripples, or charge puddles of the graphene sheet. A broad D-band having a higher intensity than that of the G-band signifies high disorderness of rGO. The G-band arises from in-plane vibrations of sp^2^-hybridized carbons, whereas the 2D-band is due to the second order Raman scattering and is particularly dominant in graphene *vis-à-vis* bulk graphite.^74^ The intensity ratio (*I*_D_/*I*_G_) of D- and G-bands can be used to quantify disorders, as represented by the sp^2^/sp^3^ carbon ratio.^73,75^Fig. 3(a) depicts the Raman spectra of rGO for varying reduction times. By employing the empirical Tuinstra–Koenig relation,^38,40,76^ relating the *I*_D_/*I*_G_ ratio and crystallite size of the graphitic sp^2^ domain, it is found that the rGO sheets embrace ordered graphitic zones having a size of 3.3 nm (for 5 h reduction treatment) enclosed by domains of oxidized carbon atoms or point defects.^38^

#### 2.2.4. XRD study

Chemical reduction was performed to convert GO to rGO and the degree of transformation was monitored by XRD studies (Fig. 3(b)). As can be seen from the figure, the peak corresponding to GO at 10° disappears after reduction and the peak at 25.6° (associated with rGO) appears.^51,52^ This peak initially remains narrow with increasing reduction time up until 3 h of treatment and then starts broadening (indicating poor crystallinity).

#### 2.2.5. UV-Vis spectroscopy

UV-Vis spectra, obtained at different reduction steps of the chemically driven sample are shown in Fig. 3(c). Two important inferences can be drawn from it – (a) the peak corresponding to absorption in the UV region is clearly visible, and (b) it is related to the π–π* transitions of aromatic C–C bonds.^51^ The gradual increase in areal intensity endorses the gradual restoration of C–C bonding with the increase in reduction time. Besides, there is a gradual red-shift of the peak at 239 nm (corresponding to GO) with the reduction time and the shift virtually saturates at the reduction time of around 5 h (267 nm).^51,52^

### 2.3. Temperature sensor fabrication

The rGO (treated for 5 h) prepared in Section 2.1 was used for temperature sensor fabrication. A self-standing film of the rGO/alumina nanocomposite was prepared *via* sol–gel processing where 0.6 wt% rGO powder was mechanically added to 99.4 wt% alumina sol as elaborated in ref. 77–79. The temperature sensor was fabricated from this film by slicing pieces of 10 mm × 10 mm size from it and depositing silver electrodes at the two end terminals of the sample.

## 3. Results and discussion

### 3.1. Charge transport mechanism

Current–voltage (*I*–*V*) characteristics of the fabricated sensor were studied in a four probe helium cryostat (Oxford Instruments) where cryo-pumping maintained the base pressure at ∼10^−7^ torr throughout the experiment. Proper care was taken to prevent stray radiation and condensation of residual moisture on the rGO film mounted inside the cryostat. The system is designed for low current measurements of the order of picoampere (pA). Current and voltage sweeps were programmed *via* LabView programme.

**Fig. 4:**
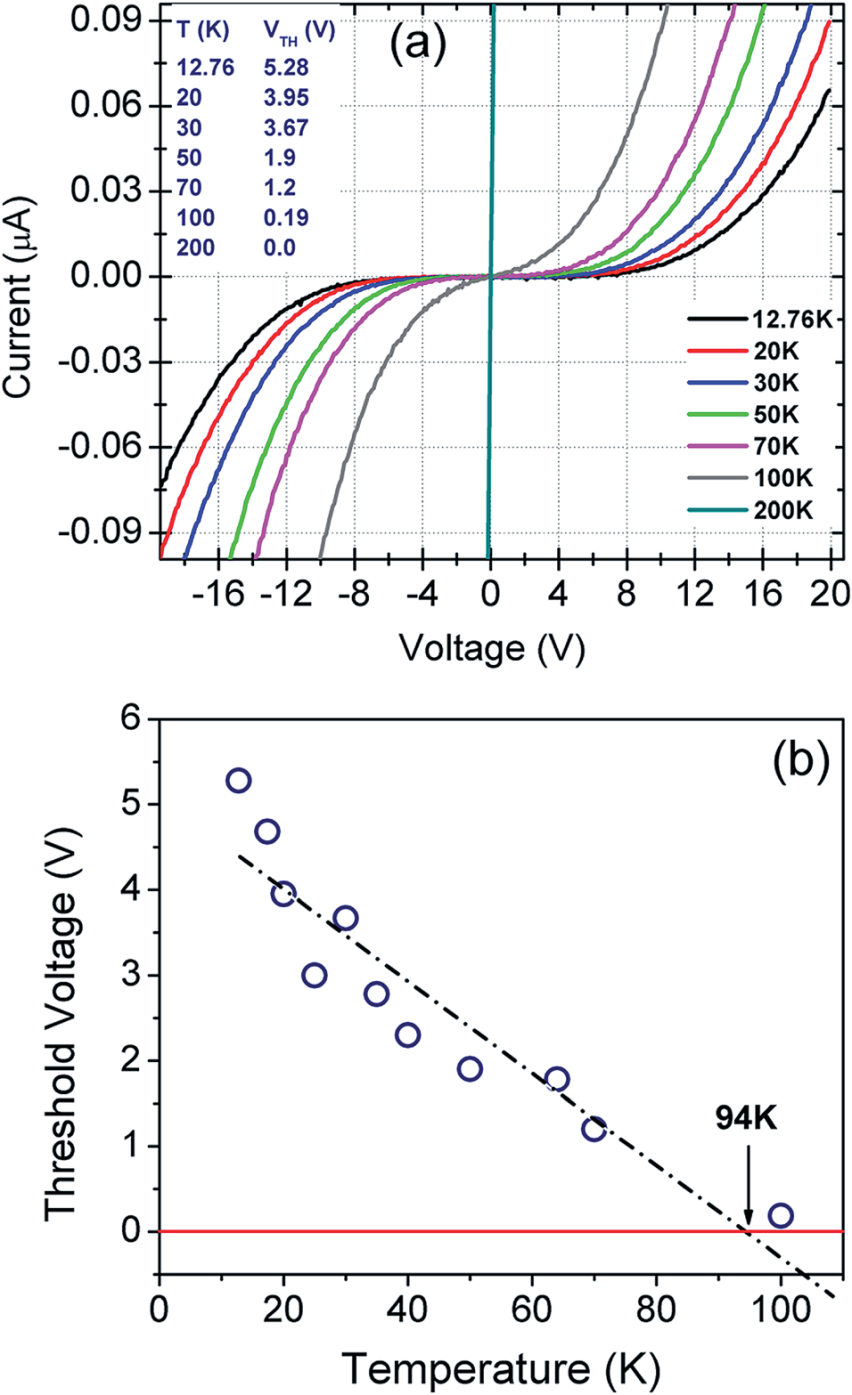
(a) Temperature dependent *I*–*V* plots of the sensor in the bias range from −20 V to +20 V. (b) Threshold voltage (*V*_Th_) as a function of temperature.

*I*–*V* measurements conducted in the 300–12 K temperature range are plotted in Fig. 4(a). The recorded plots are highly symmetric and non-linear below 100 K. Sensor breakdown was not observed even at the bias voltages of ±20 V. Low temperature (≤100 K) *I*–*V* plots indicate current suppression below a certain threshold voltage, *V*_Th_.This current suppression can be attributed to the Coulomb blockade phenomenon as extensively explained in ref. 40, 80–84. *V*_Th_ is found to be both temperature as well as bias dependent. As evident from Fig. 4(b), the threshold voltage decreases linearly with temperature up to 94 K and it becomes negative after extrapolation beyond 94 K.^80–84^ The negative *V*_Th_ has been attributed to the Coulomb blockade phenomenon by several authors.^80–84^

**Fig. 5:**
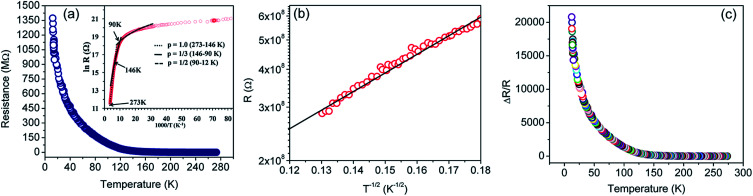
(a) Resistance (*R*) *versus* temperature (*T*) plot for the rGO sample in the 273–12 K temperature range. Inset shows *R* (in log scale) *versus* 1/*T* plot. The dotted, solid and dashed lines are fits to eqn (1) of the experimental data of (a), using *p* = 1, 1/3, and 1/2, respectively. (b) *R versus T*^−1/2^ plot in the semi-log scale. The solid black line represents linear fit for a *T*^−1/2^ behavior of the experimental points (red circles). (c) Normalized change in resistance as a function of temperature.

The resistance *versus* temperature and its semi-logarithmic plot is shown in Fig. 5. There is an anomalous jump in resistance of more than six orders of magnitude as the temperature is reduced from 273 K to 12 K. The relatively high change in conductivity *vis-à-vis* graphene is due to the presence of residual functional groups (*i.e.*, sp^3^ bonding) and disorders in rGO.^38,80–89^ Several distinct regions are clearly observed (as demonstrated in the TEM image), similar to disordered semiconductors.^80,85^

On the basis of XPS and Raman data, the rGO sheet may be considered as a two-dimensional (2D) array of GQDs where graphitic domains behave as QDs while oxygenated domains act as tunnel barriers. The temperature dependent charge-transport mechanism in rGO is generally supported by three independent theories, *i.e.* Mott variable range hopping (Mott-VRH),^38,86^ Efros–Shklovskii variable range hopping (ES-VRH),^55,87,88^ and thermal activation^52,89^ supported VRH – also known as Arrhenius type; and these are highly temperature zone specific. While looking into the theoretical aspects, there is a serious disagreement on the temperature scaling factor even for an identical temperature range; and this is because the results were obtained with different sample geometries, reduction processes, reduction temperatures and the reduction routes.^88^ Therefore, the transport mechanism in GQD arrays in rGO is an important subject in the field of graphene based electronics.

The temperature dependent resistance behavior shown in Fig. 5 probably originates from the characteristic of the strongly localized region of VRH. Mott^38,88^ theory relies on three specific criteria – (1) localized defect states are created near the Fermi level during GO to rGO conversion; and carrier transport occurs *via* hopping from one state to another by taking energy either from a phonon or directly from an externally applied electric field; (2) at low temperatures, the nearest neighbor hopping will not dominate; rather the hopping electron will always try to achieve the lowest activation energy (Δ*E*) and shortest hopping distance. Generally, these two conditions are not fulfilled simultaneously; and there exists an optimum hopping distance *r*_hop_, corresponding to maximum hopping probability; and (3) there is no Coulomb interaction between the localized states. The standard Mott-VRH relation is:^40^1
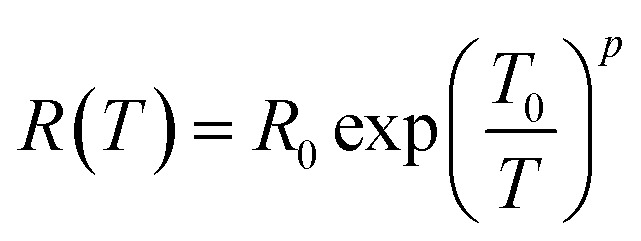
here, *R*_0_ is the resistance at zero temperature and *T*_0_ is the Mott characteristic temperature. The scaling factor *p* depends upon the dimensions of the system or the shape of density of states (DOS).^85,90^ This concept results in the mathematical expression where *R* is proportional to (*T*)^−1/3^.

Efros–Shklovskii (ES) proposed that defect states are localized around the Fermi level, transport occurs *via* hopping and there is Coulomb interaction among carriers. This idea results in *R* scaling as (*T*)^−1/2^.^40,90^2
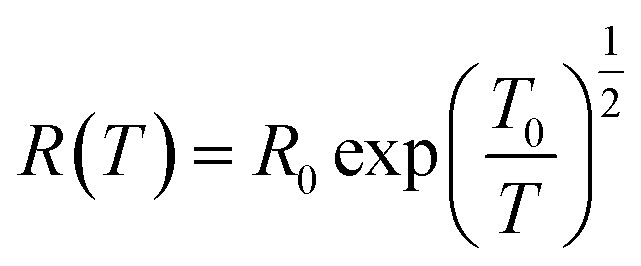


The scaling factor *p* turns out to be −1 in the case of Arrhenius theory of activation energy induced hopping and the relation is:^80^3
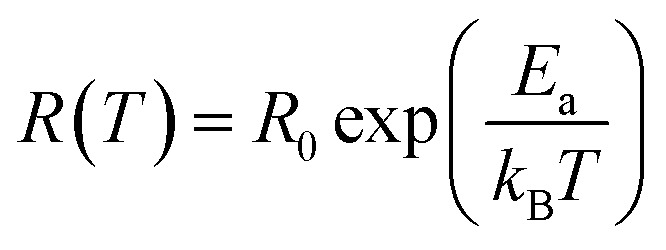


Best linear fits were obtained for 
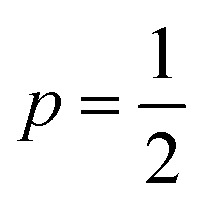
 in the temperature range of 90–12 K pointing toward ES-VRH as a plausible charge-transport phenomenon in rGO layers consisting of GQD arrays. The other fitted values are 
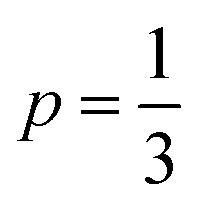
 in the 146–90 K temperature range where Mott-VRH is applicable. The temperature range of 273–146 K comes under Arrhenius type of conduction *via* activation energy where *p* = 1 is the best fit. The temperature range of 100–80 K may be a mixed zone where ES-VRH and Mott-VRH both coexist.

The ES-VRH suggests strong localization of wave functions in GQDs. Its further analysis allows the calculation of localization length *ξ* by plotting *R* against *T*^−1/2^ in a semi-log scale (Fig. 5(b)).^91–93^ From the slope of the curve, *T*_0_ = 4170 K is obtained. *T*_0_ is related to *ξ* through:^40,87^4
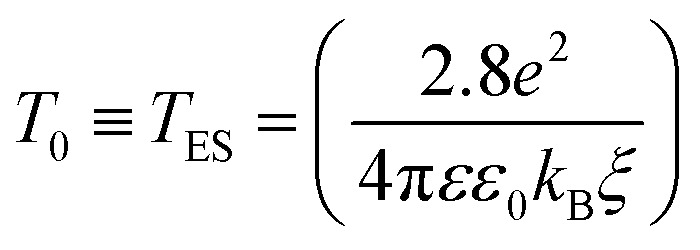
where *ε*_0_ and *ε* are the values of permittivity of vacuum and the dielectric constant of the material. *ε* in rGO is 3.5.^40^ Using eqn (4), *ξ* comes out to be ∼3.2 nm which is in close agreement with the GQD size calculated from Raman data, indicating strong localization of wave functions within graphitic domains.^40,87^Fig. 5(c) depicts the normalized change in resistance (Δ*R*/*R*) as a function of temperature. Here, Δ*R* = *R*(*T*) − *R*; *R* denotes resistance at 273 K and *R*(*T*) denotes the resistance of the sensor at a set temperature of *T* °C. The sensor exhibits an exceptionally high change in resistance leading to an ultra high TCR.

### 3.2. Temperature sensing studies

**Fig. 6:**
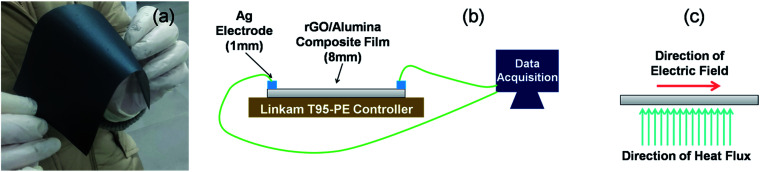
(a) Photographic image of the developed rGO/alumina composite self-standing film. Schematics of the (b) experimental setup employed to measure the temperature dependent sensor response, and (c) directions of the electric field and heat flux.

The temperature sensing measurement was conducted in a two probe system fitted with a liquid nitrogen closed cycle system (Fig. 6). The heating/cooling induced electrical response was monitored in the temperature range of 300–77 K and was recorded using a Keithley 4200 SCS. A Linkam T95-PE system temperature controller was employed to maintain the targeted temperature. Below 77 K, measurements could not be conducted due to the limitation of the liquid nitrogen cryostat.

### 3.3. TCR and hysteresis analysis

In a temperature sensor, we encounter two interdependent physical properties of the material, *i.e.* thermal (*κ*) and electrical (*σ*) conductivities; and both are responsible for heat and carrier transportation. Their interdependence determines the temperature coefficient of resistance (TCR) and thermal hysteresis loss (*H*_Th_), thereby playing an important role in efficient operation of the sensing device. A large TCR is critical for improved sensitivity, resolution, drift, and response- and recovery time.^52,77,78^

**Fig. 7:**
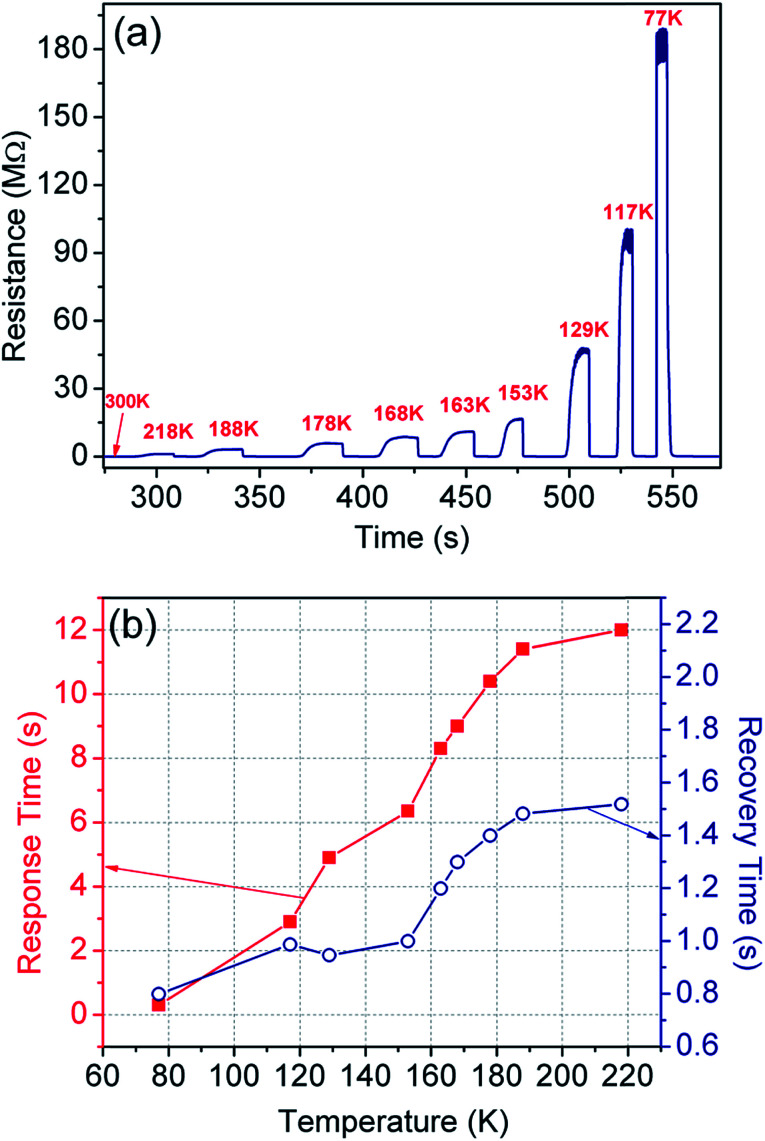
(a) Sensor operation at a specific temperature by exposing (or removing) it to a target temperature from room temperature, and (b) the corresponding response and recovery time of the sensor.

The TCR is directly calculated from the sensing measurements in non-contact, *i.e.* convection mode (Fig. 7), where the sensor was shifted from room temperature and exposed to the targeted temperature. The TCR values are tabulated in Table 1 for each temperature range along with the response- and recovery-time. The maximum TCR observed is −1999.8% K^−1^ in the temperature range of 300–77 K with a unique slope and ultra-fast response time of ∼0.3 s. Such an unprecedented increase of TCR indicates the superior performance of the sensor in terms of sensitivity and resolution. The constant slope has an added advantage that the device may not need further calibration. Further, the baseline shift is practically nil in all the cycles, therefore, thermal hysteresis does not come under purview.

**Table 1:** Sensing parameters in different temperature ranges

Initial temperature (K)	Final temperature (K)	TCR (−% K^−1^)	Response time (s)	Recovery time (s)
296	373	0.98	3.96	6.01
296	348	1.20	3.99	5.70
296	333	1.26	3.92	5.69
296	318	1.24	1.99	5.42
300	218	36.6	12.0	1.52
300	188	94.3	11.4	1.48
300	178	155.5	10.4	1.40
300	168	218.7	9.00	1.30
300	163	267.6	8.30	1.20
300	153	369.8	6.40	1.00
300	129	907.2	4.90	0.95
300	117	1753.7	2.90	0.99
300	77	1999.8	0.30	0.80

**Fig. 8:**
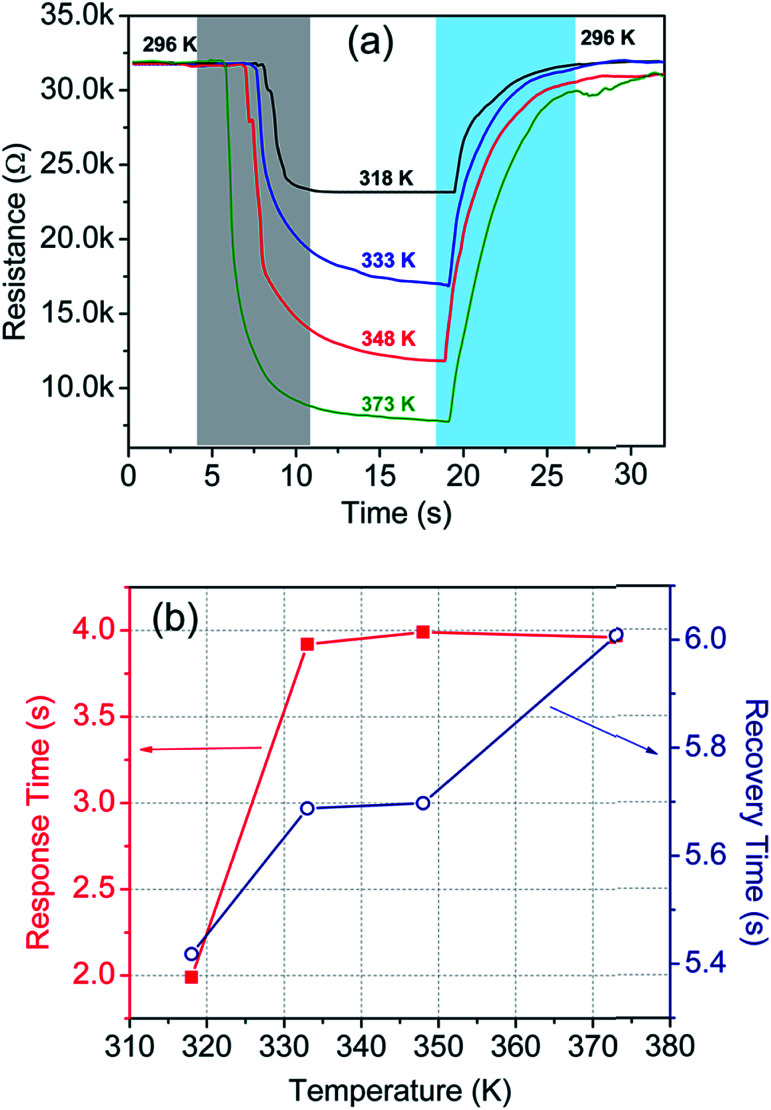
(a) Sensor response at different temperatures in the 296–373 K temperature range, and (b) response and recovery time of the sensor at the corresponding temperatures.

In addition to this, we have also carried out temperature sensing tests at high temperatures, *i.e.* in the 296–373 K temperature range. The test results are incorporated in Table 1 and plotted in Fig. 8(a). The corresponding response and recovery time are shown in Fig. 8(b).

### 3.4. Cycling test at low temperature

**Fig. 9:**
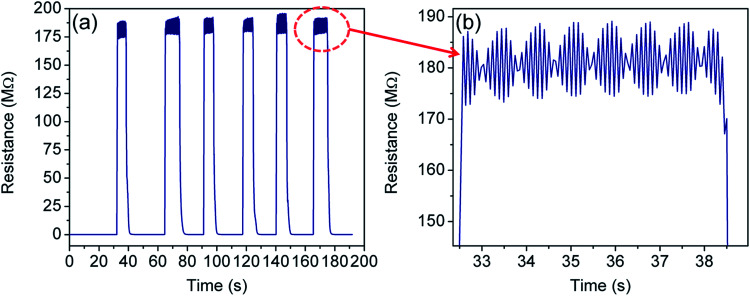
(a) Sensor response when the sensor is frequently dipped into liquid nitrogen for short time durations, and (b) zoomed in image of the highlighted spot in (a) showing the formation of the oscillatory pattern (in the form of an envelope) at 77 K.

Fig. 9 shows the cycling test of the sensor in the temperature range of 300–77 K. Excellent repeatability with a unique profile is observed in all the cycles. The response- and recovery time are found to be of the order of few seconds and shown in Table 1. Interestingly, envelope shaped oscillations appear in the sensor response. The reason for such oscillations is not clearly understood, but can be attributed to the current fluctuations when the device achieves extremely high resistance (∼GΩ) and intermittent current suppression takes place.^94^ This experiment could not be extended beyond 77 K owing to the limitation of our sensing setup which is based on closed cycle liquid nitrogen.

Interestingly, the envelope pattern resembles – (1) the superposition of the signal and carrier frequency as seen in the case of amplitude modulation in communication engineering,^95^ (2) the de-Haas van Alphen effect under a magnetic field,^96^ and (3) the superposition of a group of waves having slightly different wavelengths from each other while propagating in a medium.^97^ Notably, the oscillations of the envelope as well as the wave pattern within the envelope were found periodic and their frequency of oscillation is 0.61 Hz and 10 Hz respectively. In the present case, the first two cases can be discarded as there is neither a high frequency (carrier wave) source nor any magnetic field applied to the sensing system. Therefore, the last one is most probable and needs further explanation.

We propose these to be the Coulomb blockade effects in terms of tunneling strength between coupled dots. The type of coupling between QDs influences the character of the electronic states and the nature of transport through the QD array. Theoretical prediction supports phase transition for tunneling in coupled GQD arrays.^98–100^ The ratio of interdot separation (*D*) and size (*L*) of the QD clearly measures the coupling strength between dots. While assuming *L* as a constant, the coupling strength may be classified in terms of *D* as follows

(1) For a large *D*, there is no interaction among QDs and the CB of individual QDs is preserved, *i.e.* the number of peaks is equal to the number of energy levels within a single QD. Each peak signifies the addition of electrons to the array, one to each GQD at the same time.

(2) When *D* is the intermediate, the CB of individual QDs is lifted and a combined CB effect across the QD array materializes.

(3) For low *D*, original energy levels split in subbands; these will cross each other and the CB will be completely diluted.

Due to the formation of high density QDs, there is a possibility to have charge particle interactions between the dots and it may be supported from the second point (2) mentioned above. As a result, the de-Broglie waves of the electrons may superimpose and the oscillatory envelope function may evolve from individual to collective electronic states in a dense QD assembly.^99–104^

In our case, *D* and *L* parameters are taken as average values because GQDs were self-created as a consequence of the chemical reduction process. Therefore, the Coulomb force due to interdot coupling is affected by the dilution effect arising from high temperature as well as high bias voltage. At a high bias voltage, the charging energy becomes negligible and the envelope disappears. Similarly, when the temperature is very high, the Coulomb force between GQDs gets diluted resulting in the disappearance of the envelope.

### 3.5. Resolution test

**Fig. 10:**
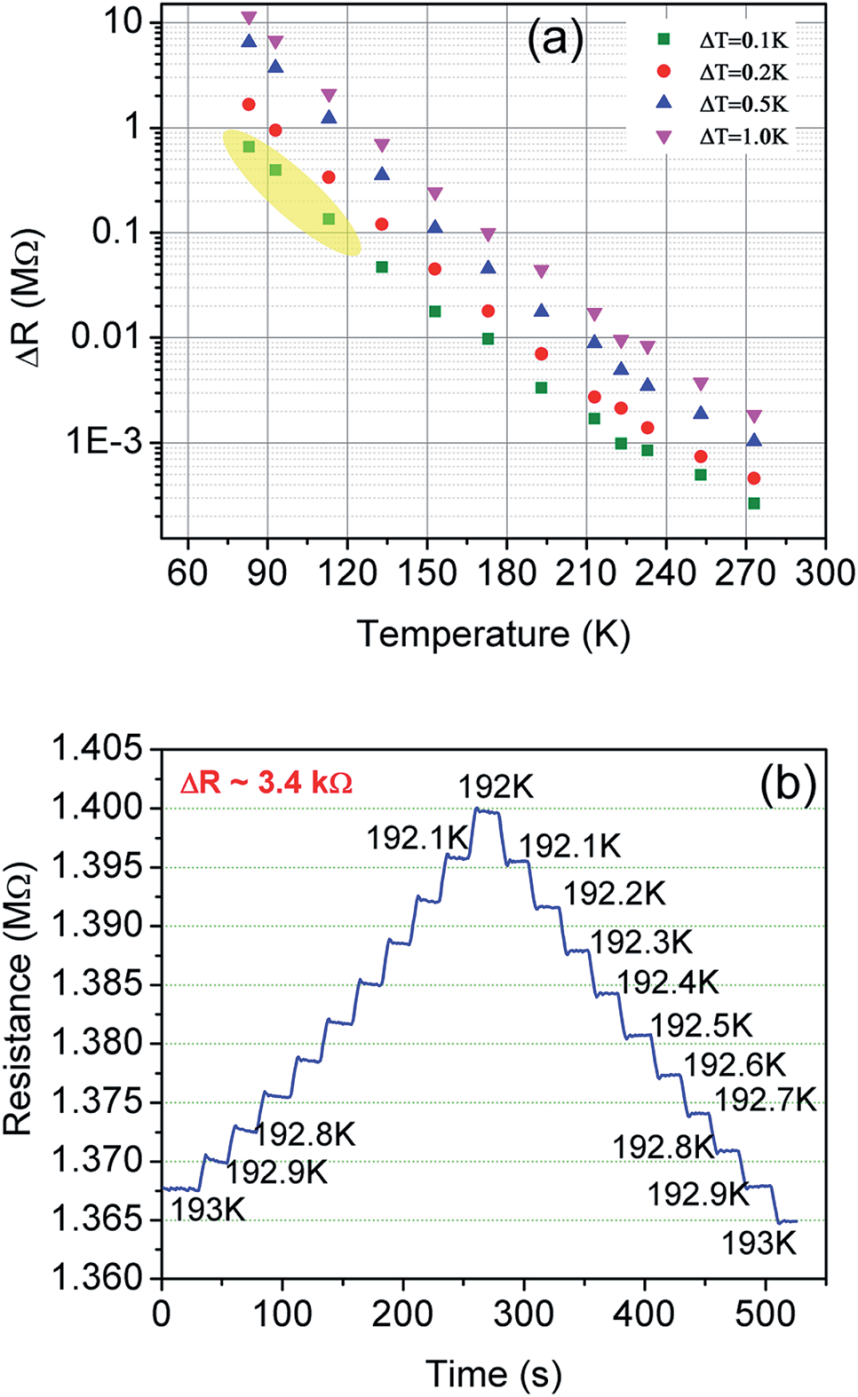
(a) Resolution test done in non-contact mode. The yellow shaded region shows the extrapolated points for the change in resistance for a 0.1 K change in temperature. (b) Shows sensor response at 193 K for a 0.1 K step change in temperature.

Fig. 10 shows the change in resistance at different temperature step sizes from 273–77 K. The temperature step size (Δ*T*) is micro-tuned to reach the targeted temperature and the corresponding change in resistance (Δ*R*) was measured accordingly. At a target temperature of 77 K, Δ*R* was found to be ∼MΩ for Δ*T* = 0.1 K (after extrapolation of the curve). For a gross simplification, if we assume the measurable signal strength to be within the 10–100 Ω range, then the signal obtained for Δ*T* = 0.1 K will correspond to a measurable temperature resolution of ∼μK. Measurements beyond 0.1 K change could not be carried out in our system due to the limitation of the temperature controller employed. As seen from Fig. 10(b), the change in resistance corresponding to a change in temperature by 0.1 K is around 3.4 kΩ indicating high resolution of the developed device.

The performance of the fabricated sensor is found to be much better than most of the existing sensors reported so far.^105–117^ A comparison is presented in Table 2 to assess its performance *vis-à-vis* some of the recently reported temperature sensors.

**Table 2:** Comparison of the sensor performance with other reported temperature sensors

Materials	Temperature range (K)	TCR (% K^−1^)	Other parameters	References
Copper oxide (CuO): pure CuO press-tablets CuO–Si-adhesive	298–353	−5.2	—	105
		−4.0		
Co-doped ZnO based thermistor	293–553	−20	—	106
Graphene nanowalls (GNWs)@polydimethylsiloxane (PDMS) substrate	308–318	+21.40	Response time = 1.6 s	107
			Recovery time = 8.52 s	
			Resolution = 0.1 K	
Reduced graphene oxide (RGO) nanosheets/elastomeric polyurethane (PU) composite	303–353	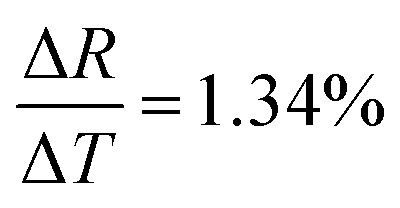	Resolution = 0.2 K	108
			Cyclability = 10 000 cycles	
Ag@polyimide based thermistor	293–333	+0.22	Thermal hysteresis = <5%	109
Pentacene/Ag NPs based organic field-effect transistor	293–373	Δ*R*/*R* = 20.4	Resolution = 0.4 K	110
Monolayer graphene based pyroelectric bolometer	Δ*T* = 0.2 K	900	Resolution = 15 μK	111
RGO decorated ZnO nanosheets	233–353	−0.205	—	112
CNT@PET substrate	233–373	−0.4	Response time = 0.3 s	113
			Recovery time = 4 s	
Multi-walled carbon nanotubes (MWCNTs)/alumina composite	303–373	−0.96	Response time = 40 s	114
			Recovery time = 185 s	
			Thermal hysteresis = 0.62%	
Poly(3,4-ethylenedioxythiophene):poly(styrenesulfonate) (PEDOT:PSS)@paper substrate based thermistor	263–298	−3	—	115
Copper oxide nanowires (CuO NWs) grown on a glass substrate	293–453	∼1.3	—	116
Vanadium pentoxide thin films	319–343	∼−4	—	117
RGO/alumina composite	300–77	−1999.8	Response time = 0.3 s	Present work
			Recovery time = 0.8 s	
	296–373	−0.98	Response time = 3.96 s	
			Recovery time = 6.01 s	

## 4. Conclusion

GQD arrays, developed in the rGO film during the chemical reduction process from GO to rGO, act as a maze where poly-dispersed graphene QDs are semimetallic, and the interdot space is an insulating zone, called the tunnel barrier. Coulomb blockade and charge carrier tunneling through the tunnel barrier is largely associated with metal and semiconductor quantum dots. The carrier transport is explained using suitable VRH models where the ESH–VRH model fits well in the low temperature regime. The average QD size or the electron localization length was found to be ∼3.2 nm. This has allowed us to translate these phenomena into making a real device, *i.e.* an ultra-sensitive low temperature thermometer with very high resolution along with the fastest response and recovery. TCR and thermal hysteresis loss are considered as the main characteristic parameters that control the quality of most of the sensor parameters and the sensor ratings. Surprisingly, both of them appeared to be exceptional in making the sensing device ultra-sensitive both in cryogenic and high temperature measurement. The cycling test revealed that the sensor is stable for over 50 cycles with an ultra-fast response time. A highly sensitive temperature sensor is in great demand for cryogenic temperature measurements in industry, healthcare, and R&D laboratories; and the proposed sensor may fulfill the need of these sectors. An interesting fact is that the sensors are cheap and reproducible. Identical rGO sheets were developed and tested successfully to justify our claim. Further, no such comprehensive report is available in the literature on the development of graphene quantum dot based temperature sensor to date.

## Conflicts of interest

There are no conflicts to declare.

## Supplementary Material
